# Rehabilitation in Amyotrophic Lateral Sclerosis: Recommendations for Clinical Practice and Further Research

**DOI:** 10.3390/jcm14238590

**Published:** 2025-12-04

**Authors:** Andreas Gratzer, Natalie Gdynia, Nadine Sasse, Rainer Beese, Cordula Winterholler, Yvonne Bauer, Carsten Schröter, Hans-Jürgen Gdynia

**Affiliations:** 1 Department of Neurology, m&i Fachklinik-Enzensberg, 87629 Füssen, Germany; 2 Clinic Hoher Meißner, 37242 Bad Sooden-Allendorf, Germany; 3 Network Dysphagia, Outpatient Center for Swallowing Disorders, 90431 Nuernberg, Germany; 4 German Society for Muscle Diseases (DGM), 97112 Freiburg, Germany

**Keywords:** amyotrophic lateral sclerosis, motoneuron disease, neuromuscular disorders, rehabilitation, neurorehabilitation

## Abstract

Amyotrophic lateral sclerosis (ALS) is a progressive neurodegenerative condition characterized by the degeneration of upper and lower motor neurons. This degeneration leads to a gradual muscle weakness, dysarthria, dysphagia, respiratory insufficiency, and, in some patients, alterations in cognitive and behavioral performance. Regardless of advancements made in pharmacological and gene-targeted interventions, a definitive curative treatment remains elusive. Consequently, rehabilitation plays a pivotal role in preserving autonomy, participation, and overall quality of life. This review outlines the current evidence and clinical approaches related to multidisciplinary rehabilitation in ALS. It covers physical and occupational therapy, respiratory, speech and language, psychological, and palliative care domains. Evidence supports moderate tailored exercise programs, early respiratory therapy, and structured management of mobility deficits, spasticity, pain, dysphagia, and communication impairments as key elements of symptomatic treatment. Psychological and social support, which includes the involvement of caregivers and relatives, enhances emotional well-being and coping resilience. Even with progressive development of gene-targeted and disease-modifying therapies, rehabilitation will stay relevant for maintaining long-term motor function. This review highlights the need for standardized, evidence-based rehabilitation protocols and intensified neurorehabilitation research to strengthen clinical outcomes and quality of life as key therapeutic goals in ALS management.

## 1. Introduction

Amyotrophic lateral sclerosis (ALS) is typically characterized by the degenerative loss of upper and lower motor neurons, which leads to weakness of the bulbar, limb, thoracic, and abdominal muscles [1]. In the course of the disease, mobility deficits, dysarthria, dysphagia, and respiratory insufficiency occur. Cognitive impairments and behavioral abnormalities are also observed in 50% of patients [2,3].

### 1.1. ALS and Its Impact on (Daily) Life

Life expectancy after disease onset is reported to be 2 to 5 years, mostly due to respiratory failure. The European guidelines [3] report a median of 3 years after disease onset. However, 5–10% of patients survive a decade or more [1].

As symptoms progress, the capability of performing activities of daily living, such as mobility, self-care, communication, feeding, and household activities, becomes increasingly difficult. Numerous reviews report that ALS affects nearly all domains of daily functioning, including dressing, bathing, walking, transferring, and maintaining social roles [4,5,6]. Declining functional abilities are strongly associated with reduced participation, loss of independence, and increasing caregiver dependence [4,7]. These changes, combined with declining engagement in valued social roles, contribute significantly to reduced quality of life [4,5].

### 1.2. The Role of Rehabilitation in ALS Management

Since there is no existing cure for ALS, symptomatic treatment is of utmost importance. Current management relies on symptomatic and supportive interventions. Numerous reviews emphasize that multidisciplinary rehabilitation significantly improves quality of life, reduces disease burden, and supports survival [4,5,6]. Therefore, palliative care is required from the onset of the disease; as Leo McCluskey [8] stated, “The palliation of ALS begins with breaking the news and ends only at the end of life”. While ALS patients were considered “untreatable” in the past, they now clearly benefit from a multifaceted interdisciplinary symptomatic treatment, in terms of survival and quality of life [4,5,8].

In recent years, there has been growing progress in the development of causal and molecular genetic therapies for ALS. Gene-based therapies, such as tofersen for SOD1-associated ALS, are being increasingly studied and implemented in clinical practice [9]. Although these emerging treatments may extend survival, they cannot cure the disease. Such novel approaches may lead to improved disease outcomes and prolonged survival in the future. Rehabilitation will, therefore, remain fundamental to maintain autonomy, participation, and quality of life, especially as patients live longer and face prolonged disease progression. Since narrative and systematic reviews consistently identify rehabilitation as a core component of ALS management [6,10,11], the advancing therapeutic landscape highlights why a comprehensive review of rehabilitation in ALS is of major clinical relevance.

The main goal is to optimize the patients’ function, autonomy, and quality of life by targeting a wide range of patients’ needs, including the relief of symptoms such as mobility deficits, spasticity, pain, and fatigue. Maintaining functional performance is supported through programs that focus on functional training, flexibility, and range of motion, and in earlier stages, moderate and adapted strength training. Another essential component is psychological care. It should be provided to address emotional effects such as depression, anxiety, and grief. This approach contributes to an overall improvement in quality of life [6,7].

### 1.3. Goal and Objective of This Review

Although individual rehabilitation domains (e.g., physical therapy [12], respiratory care [11], and caregiver support [10]) are discussed in reviews, to our knowledge, there is no current review covering physical, occupational, respiratory, speech-language, psychological, and palliative interventions collectively. Therefore, this review aims to provide an overview of current rehabilitation strategies for ALS. It covers the areas of physical, occupational, respiratory, speech, and psychological therapies, in the frame of palliation, tailored to the needs of ALS patients. The purpose is to aid clinical decision making and guide healthcare providers toward more standardized and evidence-based rehabilitative care for this complex and progressive disease.

## 2. Multidisciplinary Care in ALS Rehabilitation

### 2.1. Composition of a Multidisciplinary Approach and Its Benefits

A multidisciplinary team in ALS care usually consists of neurologists, physiotherapists, speech and language therapists, respiratory therapists, occupational therapists, psychologists, and specialist nursing staff, as well as nutritionists and social workers [13]. Each profession contributes, with specialized expertise, to meet the wide-ranging and complex needs of the patients. Voluntary support services, most often in the form of national or regional ALS associations or self-help groups, also make a significant contribution to patient care [13]. Further studies [4,5] support these findings, demonstrating that multidisciplinary care improves symptom control and enhances quality of life.

The collaborative nature of multidisciplinary care supports a comprehensive and patient-centered approach. Team members work closely together to develop and implement individualized treatment plans that address physical, emotional, and social needs. Regular communication through interdisciplinary team meetings guarantees that patients receive well-timed, coordinated, and effective care. This approach not only contributes to improving and stabilizing functional outcomes, including mobility, respiratory parameters, communication abilities, nutritional status, and psychological well-being, but also increases life expectancy in ALS patients. Reviewers consistently show that multidisciplinary interventions slow decline in ALS Functional Rating Scale Revised scores (ALSFRS-R) [4,5,6,11].

#### 2.1.1. Symptomatic Therapy of ALS

For nearly 30 years, riluzole has been approved in Europe. As a recent study has shown, riluzole prolongs life expectancy after diagnosis by an average of approximately seven months, particularly in patients with bulbar onset or rapid disease progression [14].

In about 20% of patients with ALS, an underlying genetic mutation can be found [3]. For certain mutations, therapy studies are being performed. So far, only one gene therapy with the drug tofersen has been approved for patients with SOD1 mutation (fALS1) [15].

Since there is no cure for the disease, symptomatic treatment and palliative care play a crucial role. McCluskey [8] describes this approach as an ongoing palliative process from the time of diagnosis until the end of life.

The German guidelines [16] recommend a combined approach of causally oriented, specific pharmacological therapy, as well as palliative medical care, including symptomatic therapy and providing appropriate assistive devices for patients. Active and early treatment should be pursued in specialized neuromuscular clinics by experienced therapists working within an interdisciplinary team due to the rarity of the disease [16]. Among the goals of symptomatic and palliative therapy, maintaining autonomy and quality of life should be prioritized [4,7,16].

#### 2.1.2. Counselling

The German guidelines [16] also emphasize the importance of early counselling after confirmation of the disease. Patients should be informed early on regarding the possibilities and limitations of non-invasive and invasive treatment options concerning nutritional support, ventilation therapy, and symptomatic and palliative medication options. They also advise setting up an advance directive at an early stage to provide guidance for health care providers and family members regarding preferred future treatment options [16]. This advanced decision making should be revised every few months [8,17].

### 2.2. Symptom Management

#### 2.2.1. Training and Mobility in ALS Rehabilitation

In rehabilitation, in general and in physiotherapy specifically, the goal is to improve or stabilize function and activities. These include trunk stability, transfers, and maintaining upright postural control in stance and gait. Exercises involving coordination tasks contribute to improving postural stability and endurance, maintaining mobility and participation in daily activities [18,19,20,21]. Moderate, supervised exercise is considered safe, with several reviews noting the absence of adverse effects when intensity is tailored to patients’ capacity [12,21,22,23,24].

Mobility impairments significantly reduce the quality of life for both patients and caregivers. Therefore, rehabilitation in ALS should include exercise programs, adapted to the patients’ current physical capacity. These programs should be developed with therapists experienced in the disease, considering the type and degree of physical intensity. As shown in studies by Bello-Haas et al. [18], Drory et al. [19], and Ferri et al. [20], tailored exercise programs can help maintain motor function.

Kudritzki and Howard [22] emphasize that when prescribing exercises, overexertion must be avoided. Furthermore, training intensity should be moderate and adapted to the patient’s capacity, with special caution for muscles unable to move against gravity, whereas eccentric strengthening should be avoided. In addition, aerobic training is considered beneficial. Moreover, to ensure safety and appropriateness, supervision by experienced physiotherapists is emphasized. Finally, where specialized ALS centers are not easily accessible, telemedicine-based training may be an attractive option, though evidence remains limited.

Fenili et al. [23] conducted a narrative review summarizing preclinical and clinical studies on exercise interventions in ALS. They considered the strength of evidence concerning previous studies on training in patients with ALS as limited. They specifically pointed out that heterogeneity and retrospective study designs were limiting factors. Furthermore, resistance training sometimes improved muscle strength. However, it did not slow down disease progression. In addition, the effects of endurance training were inconclusive. In contrast, mouse model studies of the disease showed positive effects. Notably, exercise programs involving treadmill training, or, even more so, swimming, were able to improve motor function and, in some cases, even survival duration. Nevertheless, whether these results can be transferred to humans still needs to be examined in appropriate studies.

Angelini and Siciliano [21] reported in their systematic review and meta-analysis that most clinical studies suggest that resistance training and endurance training can improve quality of life and functionality and, in some cases, improve their cardiorespiratory function and/or muscle strength. They also reported that the findings of most studies on patients with ALS still appear preliminary and, therefore, limit conclusions due to inadequate sample sizes and inadequate control of variables, including lack of representative control populations and varying ALS disease stages. Moreover, they emphasized that exercise programs should minimize patients’ risk of falling. Finally, exercise programs with high resistance and eccentric training should be avoided.

In a recent review on muscle strengthening in ALS patients, Souza et al. [12] found that seven out of eight studies favored experimental interventions over controls; however, this was not supported by meta-analysis. Evidence quality was low due to small sample sizes and high heterogeneity among intervention protocols. Strengthening interventions did not show superior short-term efficacy but appeared safe, with no serious adverse events mentioned. In a related review, Silva et al. assessed randomized controlled trials of physiotherapeutic interventions including stretching, resistance, aerobic, and aquatic training, as well as relaxation and self-management/energy-conserving strategies. The interventions showed minor short-term functional benefits but had no impact on quality of life or fatigue, again with no adverse events reported [12,24].

A novel approach to evaluate the effects of exercise programs by performing transcriptome analysis was conducted by Jawdat et al. [25]. In a pilot study including seven early-stage ALS patients, completing a 3-month quadriceps-focused home program, transcriptome analysis of material from needle biopsies of the trained muscles before and after the exercise program was performed. They found that clinical outcomes, muscle strength, functional status, and ALSFRS-R remained stable. However, gene activity increased in genes that are typically reduced in ALS. Whether this has clinical relevance for the course of the disease still requires confirmation with a larger cohort and over a longer period [25].

Overall, most studies were conducted focusing on strength and endurance training, as these can be well standardized. From the available literature [18,19,20,21,23], it can be concluded that with moderate, individualized implementation, a deterioration in function and strength is not to be expected. Thus, exercise programs can be recommended under the principle primum nil nocere. Overexertion should be avoided. Warning signs may include increased fatigue or a rapid reduction in walking distance. It should also be noted that in rehabilitation clinics, the walking distance within the clinic is often longer than those usually covered at home, and in combination with therapeutic interventions, may be unfamiliar for patients.

#### 2.2.2. Occupational Therapy

Occupational therapy in ALS aims to maintain independence, autonomy, and quality of life throughout disease progression. By identifying patient-specific challenges that arise from progressive functional motor decline, occupational therapy focuses on developing strategies to overcome these obstacles, enabling patients to participate in meaningful activities and maintain a sense of control over their lives [26]. Interventions focus on maximizing remaining motor function and adapting activities of daily living through individualized strategies, including energy conservation strategies, cognitive training exercises, and adaptive equipment. This may involve recommending assistive devices, splints. and environmental modifications that enhance safety and independence in daily activities, while supporting the patient’s ability to perform essential tasks with greater ease and efficacy [26,27].

For the upper extremities, occupational therapy is applied with similar goals as physiotherapy. Both disciplines emphasize assistive device provision [26,27]. A lot of ALS patients struggle to accept mobility aids at first, especially wheelchairs [27]. In inpatient rehabilitation, different wheelchair models can be tested and implemented, making it easier to make appropriate adjustments for current symptoms and future needs. However, health insurance providers often initially reject these specialized and costly devices, leading to months of delay. Such delays supporting mobility significantly affect activities, participation, and the quality of life of patients and caregivers.

#### 2.2.3. Speech and Language Therapy

In the interdisciplinary management of ALS, speech and language therapy (SLT) plays a key role in maintaining quality of life by addressing communication and swallowing disorders [10]. Due to the disease mechanism of ALS, a purely rehabilitative therapy that focuses exclusively on functional improvement is not effective. However, this does not mean that the care of people with ALS should fall into therapeutic nihilism [3,6,8,16]. The core goals of SLT are as follows:

- Preserving communication ability for as long as possible through early education on strategies [3,28].
- Enabling safe oral intake for as long as it is desired/feasible through continuous screening, counselling, and integration of adaptations, such as Smooth FOOD, “foam”, etc. [29,30].
- Supporting informed decisions about nutritional pathways through education on indications, benefits. and timing of PEG during disease progression [3,31,32].
- Use of EDAR (eating and drinking with acknowledged risks) when intake is desired despite known risks [17,33].
- Saliva management through counselling on individualized measures (e.g., positioning, suctioning of secretions, and oral hygiene) in coordination with medical/pharmacological treatment [34,35].
- Strengthening participation, autonomy, and quality of life through individual, everyday-oriented goal setting, involvement of caregivers, advance-care planning, and regular re-evaluation [3,8,16].
- Regular monitoring of swallowing ability in relation to breathing and cough strength through longitudinal follow-up and timely adaptation of interventions and assistive devices [3,16,35].

##### Dysarthria and Anarthria

Dysarthria and anarthria are among the earliest and most common symptoms of ALS patients, affecting over 80% during the disease course. Impacts on articulation, prosody, phonation, and breath–speech coordination often lead to anarthria. This is particularly evident in bulbar onset, where disease progression advances quickly, and the prognosis is less favorable [36,37,38]. SLT diagnostic assessments include clinical, perceptual, standardized, and instrumental methods as well as patient-reported protocols. Lee et al. [28] examined clinical assessment tools for dysarthria and evaluated their effectiveness regarding diagnostic validity, functional relevance, and feasibility in everyday clinical use. The assessments were divided into three categories: acoustic measures (e.g., loudness, pitch, and speech rate), kinematic measures (movement of tongue, lips, and jaw), and strength measures (e.g., tongue strength). No single method fully meets all criteria, although some appear diagnostically promising [28]. The EAN guideline [3] emphasizes early SLT involvement, dysarthria-focused interventions, and structured management within multidisciplinary ALS centers. Regular documentation is essential due to the progressive nature of dysarthria and its impact on therapeutic decisions. Wolfrum et al. [39] highlighted potential issues with the fact that therapists often rate their own patients more favorably. These judgements may be influenced by familiarity with the patient and therapeutic bias [39], underlining the need for objective, independent assessment methods. Evidence suggests that strengthening or restorative exercises are ineffective and potentially harmful in ALS [38]. Instead, compensatory strategies are favored, such as deliberately slowing speech rate or using short phrases to improve speech clarity [40]. Thus, maintaining communication and preparing patients for augmentative and alternative communication (AAC) are key therapeutic goals. The primary goal of AAC is to maintain communication and social participation despite progressive loss of speech. ACC includes low-tech methods such as symbol boards and high-tech solutions such as speech-generating devices, tablets, and eye-tracking systems [41,42]. Early implementation is recommended to ensure a smooth transition from verbal to alternative communication and prevent frustration and/or social isolation [43,44]. Newer innovations such as artificial intelligence (AI), text prediction, and smart-home integration offer promising possibilities to enhance participation and quality of life for people with ALS [45]. Further research in this field should involve end users and focus on usability, accessibility, and cost effectiveness. The first pilot studies on personalized AI-generated voices have been conducted with increasing interest. This could also be promising for patients with limited speech [46].

##### Dysphagia

Dysphagia is a frequent and clinically significant complication in ALS, particularly in bulbar onset, where it occurs in an early disease stage [47]. Regardless of the ALS phenotype, up to 70–80% of patients are affected. This leads to risks of malnutrition, aspiration pneumonia, and mortality [48]. Early diagnostics and interprofessional management are, therefore, essential. Impairments involve tongue strength, velum function, and airway protection [28,39]. This results in oral residues, nasal penetration, and delayed or insufficient bolus transport, which leads to an increased risk of aspiration [49,50]. Clinical screening assessments such as the ALSFRS-R, Eating Assessment Tool (EAT-10), as summarized by Lee et al. [28], and the Swallowing Quality of Life Questionnaire (SWAL-QOL) [51] support detection, whereas fiberoptic endoscopic evaluation of swallowing (FEES) and videofluoroscopic swallow study (VFSS) remain the gold standard [52]. Therapeutic approaches include supportive strategies, such as texture modification according to the International Dysphagia Diet Standardization Initiative Framework (IDDSI), postural adjustments like the “chin-tuck”, and caregiver training [29,30]. There is limited evidence for rehabilitative interventions like expiratory muscle strength training (EMST) [53,54]. Neuromodulate approaches such as pharyngeal electric stimulation (PES) have not shown benefits [55]. Nutritional management is critical and associated with improved prognosis when performed early [31]. PEG insertion should be considered early, before respiratory weakness occurs. Guidelines recommend PEG insertion in cases of weight loss greater than 10% or when the ALSFRS-R swallowing score is ≤2 [3].

##### Eating and Drinking with Acknowledged Risks (EDAR)

EDAR is the decision to continue oral intake despite dysphagia and risks involved, prioritizing autonomy and quality of life within a structured and ethical process [33]. EDAR requires early and ongoing counselling on nutritional options, including texture modification, enteral feeding, or PEG [16,32]. Key components are clarifying patient values, transparent risk–benefit communication, and regular re-evaluation [17,32]. EDAR supports informed decision making and individualized strategies and avoids therapeutic nihilism. For SLT, it means a shift from solely focusing on aspiration prevention to enabling patient choice and participation [48].

##### Caregiver Involvement in Speech Language Therapy for ALS

Caregiver involvement is a big part of SLT in ALS, as family members are often involved in communication, feeding, and daily care. Research shows that interventions are more effective when caregivers are involved in training and strategy adaptation [56,57]. Joint interventions increase motivation and comprehensibility by helping partners adapt to changed speech patterns [45,58]. Key areas are communication support, dysphagia management, and psychosocial support [33,48,59]. Guidelines recommend caregiver involvement to increase participation and quality of life [3].

#### 2.2.4. Respiratory Impairment and Secretion Mobilization and Clearance

ALS leads to a progressive paralysis of skeletal and respiratory muscles, followed by difficulties in inspiration and expiration [60]. In rare cases, respiratory insufficiency can be an initial symptom [61] but usually occurs later in the course of the disease. Respiratory insufficiency plays a decisive role in the progression of the disease and is the leading cause of death in ALS. Patients die from hypercapnia caused by alveolar hypoventilation, leading to CO_2_ narcosis and hypoxia [60,62]. Therefore, respiratory dysfunction and its associated symptoms play a major role in the symptomatic treatment and rehabilitation of ALS.

##### Respiratory Therapy

The aim of respiratory therapy in the early stages is to strengthen respiratory and accessory muscles through tailored interventions. Core elements include breathing awareness training, airway secretion clearance, assisted coughing, and chest mobilization [11]. Breathing exercises such as resistance breathing (in early stages), passive stretching, and breathing-relief posture training are applied [11,60]. The implementation of respiratory therapy as part of symptomatic treatment in ALS is recommended by the German AWMF guideline on motor neuron diseases [16]. In terms of the evaluation of evidence on respiratory therapy in ALS, studies are limited. Some studies showed modest or no lasting benefits [63,64,65]. However, inspiratory training with an electronic device did improve inspiratory pressure and heart rate variability in other studies [66,67]. A more recent randomized, multicenter, sham-controlled trial showed that mild respiratory training improved maximal expiratory pressure and cough peak inspiratory flow and slowed bulbar functional decline, providing class II evidence for its effectiveness in early-stage ALS [53].

##### Improving Cough Efficiency

Coughing is an essential protective mechanism to clear excessive secretion or foreign materials from the airways. This requires coordinated action of inspiratory, expiratory, and glottic muscles [11]. Progressive muscle weakness in ALS reduces cough strength, significantly affecting quality of life [60]. Particularly in patients with bulbar symptoms, the glottic closure becomes ineffective, which further compromises cough efficiency. Respiratory muscle weakness and impaired cough efficiency consistently result in bronchial secretion build-up, which presents a high risk for respiratory infections [11]. When dysphagia is present, insufficient cough clearance is especially problematic. This makes the improvement of cough strength an important therapeutic goal in rehabilitation [11]. Supportive strategies such as pharmacological, manual therapeutic, and device-based interventions are considered when addressing secretion management [11,16].

##### Secretion Management

Pharmacologically, anticholinergics that inhibit muscarinic receptors are used to reduce hypersalivation in ALS. Common agents include N-butylscopolamine, mostly given as a transdermal patch, sublingual atropine drops, and sustained-release amitriptyline [60]. According to the German Society for Otorhinolaryngology, head and neck surgery guidelines for hypersalivation, pirenzepine, and glycopyrrolate are also frequently prescribed in Germany, while the international literature further reports the use of ipratropium bromide and trihexyphenidylbenzhexolhydrochloride [34]. Evidence concerning the use of anticholinergic agents in ALS is scarce. There are only a few clinical studies available [35,68], and the German guideline on hypersalivation notes the lack of robust data [34]. The choice of drug and its dosage should be determined individually for each patient. Sublingual or transdermal dosage forms are preferred in advanced disease, since not all forms of oral agents are suitable for percutaneous endoscopic gastrostomy (PEG) administration [35]. Data from the ALS-Care-Base show that more than half of patients respond to atropine, glycopyrrolate, or amitriptyline [69]. A further study reported an efficiency of 85% for transdermal-applied patches. However, in some patients, monotherapy was insufficient, and in 20%, the patch had to be discontinued due to skin reaction during treatment [70]. Overall, anticholinergic agents represent the first-line pharmacological therapy for the management of hypersalivation in ALS.

Mucolytics such as N-acetylcysteine may be useful when cough strength is sufficient or in combination with an insufflator–exsufflator device, but are otherwise contraindicated [1]. Secretolytics like ambroxol can enhance mucociliary clearance, though they should only be used when adequate cough function remains [71].

Gruti et al. [35] note in their review that injections with botulinum toxin into salivary glands provide an additional therapeutic option. However, they emphasize that evidence is limited due to the heterogeneous protocols across the studies. Furthermore, the authors report that radiotherapy and surgical interventions are also given as options.

Core manual therapeutic techniques such as postural drainage, assisted abdominal thrusts, and air stacking can support secretion clearance and improve cough efficiency in ALS [60,72,73]. The physiotherapy literature emphasizes the value of individualized approaches, which are considered a low-cost first-line option [72].

Effective device-based interventions include insufflation–exsufflation (“cough assist”) to support cough efficiency and improve pulmonary clearance [74]. Furthermore, vibratory methods such as high-frequency chest wall compression and intrapulmonary percussive ventilation may also aid secretion mobilization [75]. The use of insufflation–exsufflation devices can reduce infection and hospitalization rates in patients with ALS [76,77].

##### Laryngospasms

In laryngospasms, the pathology refers to spasms of the vocal cord muscles, which may cause dyspnea, anxiety, and a sensation of suffocation. They occur in various neuromuscular diseases [78]. For example, in Kennedy-type spinal and bulbar muscular atrophy, they affect over 50% of patients and a smaller proportion of ALS patients [78]. If laryngospasms are present, upright posture, postural support, and slowed breathing are recommended [79]. Prevention focuses on reflux treatment and avoiding large meals. In patients with known laryngospasms, antisecretory and prokinetic agents are recommended [78,79]. In some selected cases, botulinum toxin injections into the vocal cords may be considered [80].

##### Ventilation in ALS

Respiratory function is a crucial prognostic factor in ALS, demanding regular monitoring of respiratory symptoms and pulmonary function [16]. Thorough medical history taking, including questions on dyspnea, hypoxia, hypercapnia, and nocturnal hypoventilation, fragmented sleep, or morning headaches, should be performed regularly [16,61]. Instrumental assessments are essential and should include measurements of vital capacity, nocturnal capnometry, oximetry, sniff nasal inspiratory pressure (SNIF), and peak cough flow (PCF) [16,81,82].

##### Non-Invasive Ventilation (NIV)

The German pneumological guideline recommends that NIV should be implemented when symptoms of sleep disordered breathing or evidence of significant respiratory muscle weakness are present. In addition, at least one of the following criteria should be present: daytime hypercapnia (pCO_2_ > 45 mmHg), nocturnal hypercapnia (tcCO_2_ or pCO_2_ > 50 mmHg), a nocturnal increase in tcCO_2_ or pCO_2_ of more than 10 mmHg above the baseline or daytime value, or a rapid decline in vital capacity during the disease course [60,83]. However, the German guideline on motor neuron disease points out that, due to the absence of supporting studies, clinical findings and patient symptoms should guide the indication [16].

NIV can improve symptoms of respiratory insufficiency as well as quality of life, with moderate evidence showing a survival benefit of about 48 days [84], and in the subgroup with better bulbar function, a median survival benefit of 205 days with maintained quality of life for most of this period [85]. Experienced physicians are essential for the initiation and adjustment of ventilation, particularly in bulbar patients [16,86].

##### Invasive Ventilation

Invasive ventilation should be openly discussed with all patients during the disease course. In patient consultations, the full extent should be explained in a detailed and honest manner, and, where possible, together with their relatives [87]. Possible complications and end-of-life issues must also be addressed. Long-term ventilated patients usually die from respiratory infections or complications rather than respiratory insufficiency. Both NIV and invasive ventilation require informed consent, which can be withdrawn at any time. Following detailed counselling, ventilation may be discontinued with appropriate medication to allow a dignified death without dyspnea or pain [88]. Emergency intubations without prior counselling should be avoided. Therefore, advance directives covering intubation and ventilation are strongly recommended [16]. If invasive long-term ventilation is explicitly requested, it should be initiated according to established principles. In tracheostomy management, the focus is on preserving quality of life, for example, by aiming to facilitate intermittent speech through cuff deflation during ventilation pauses, rather than weaning. Several techniques and communication aids are available to facilitate speech, but evidence for their effectiveness remains poor [89].

#### 2.2.5. Pain and Spasticity

Pain is an often underestimated and neglected symptom in ALS. While the disease is primarily defined by atrophic and spastic paralysis due to damage to the upper and lower motor neurons, recent research increasingly highlights pain as a significant yet underestimated aspect. Several studies report a high prevalence of pain, though exact numbers vary. Hanisch et al. [90] found that 78% of ALS patients experienced pain. A meta-analysis by Hurwitz et al. [91] estimated an average prevalence of about 60% across 21 studies. Considerable heterogeneity (I^2^ = 94%) among the 21 studies indicates methodological differences such as varying assessment tools used, disease stage, and pain definitions. Pain substantially affects daily life, mood, and enjoyment of life [90] as well as social relationships, sleep, and mobility [92]. Furthermore, pain intensity correlates positively with reduced quality of life, highlighting its significant impact on overall well-being in ALS [90,92,93,94].

##### Pain Evaluation

Despite the high prevalence of pain in ALS, there are currently no specific guidelines for routine pain screening established across clinical practice [1,95]. In a survey of U.S. American ALS clinics, 92% of physicians reported routinely assessing pain, mostly through open-ended questions. Less than 20% used standardized instruments [96]. The Brief Pain Inventory (BPI) is the most frequently used tool, followed by other validated assessments, such as the Neuropathic Pain Scale and the McGill Pain Questionnaire [91]. The diagnostic value of these assessment tools is, however, limited, as pain perception is strongly influenced by psychological factors, including attention, context, and cognitive processing [97,98].

##### Pain Characteristics

Pain in ALS occurs with diverse characteristics and underlying mechanisms. Nociceptive pain is most common, often resulting from muscle atrophy, joint stiffness, immobility, or pressure ulcers. It is typically described as aching, cramping, or sharp [92]. Spastic pain and cramping are further characteristic features of ALS. Cramps represent one of the most common and distressing sources of pain, often movement-induced and predominantly affecting distal muscle groups such as calves, thighs, hands, and feet, often occurring at night [90,94,99]. In a longitudinal study, muscle cramps occurred in up to 95% of patients, with 24% reporting severe pain, particularly in spinal onset [100]. Electrophysiological evidence indicates that cramps in ALS originate from instability of motor units at distal motor nerves and from muscle denervation [94].

Spasticity, present in 11–36% of patients, arises from impaired suprasegmental control and spinal inhibitory dysfunction, leading to painful muscle stiffness and contractions [94]. A further important aspect is neuropathic pain, often described as burning, tingling, allodynia, and hyperalgesia in ALS, typically with distal or diffuse distribution [94,99]. However, findings are discussed controversially. While some studies report neuropathic pain, others argue that diagnostic criteria are often not fulfilled in ALS [1,92]. Reported prevalence rates range between 6,9% and 10%, comparable to the general population [101,102]. A potential contributing factor to this low prevalence may be the use of riluzole, which, in experimental rodent models, demonstrated analgesic effects through modulation of NMDA receptors, inhibition of microglial activation, and activation of TREK-1 channels [103,104]. In humans, however, clinical studies have shown no significant analgesic effect of riluzole in the treatment of peripheral neuropathic pain [105].

##### Pain Progression in ALS

The time course of pain in ALS and its association with disease severity remain uncertain. In a population-based controlled study, Chiò et al. [106] found that pain frequency and intensity were correlated with lower functional scores and longer disease duration. This suggests that pain may increase as mobility decreases. In contrast, Hanisch et al. [90] and Rivera et al. [107] did not confirm this relationship. Hanisch et al. observed no correlation between disease duration and pain severity, while Rivera et al. reported that pain was present in all stages of ALS. Pain intensity correlated only weakly with functional status. Together, these findings indicate that pain can occur throughout the disease course. These inconsistent findings highlight the need for individualized diagnostic and therapeutic approaches as well as further research [90,106,107].

##### Pharmacological and Non-Pharmacological Pain Management

Pain management in ALS is mainly based on pharmacological treatment, often combined with non-pharmacological interventions [99]. Various medications are used to relieve pain in ALS, and drug selection should be guided by the underlying cause and type of pain [94].

1. Pharmacological management

(1) Nociceptive pain

Non-opioid analgesics such as NSAIDs (nonsteroidal anti-inflammatory drugs) and paracetamol are commonly used as first-choice therapy for nociceptive pain, reducing musculoskeletal discomfort and improving mobility through anti-inflammatory mechanisms [108]. In cases of localized joint pain, intra-articular corticosteroid injections may be beneficial, either alone or in combination with lidocaine [94]. Non-opioid drugs are the most frequently prescribed drug class, used in 54% of ALS patients experiencing pain [109].

Opioids are not considered first-choice agents in ALS. They are typically used when non-opioid analgesics fail to provide sufficient relief. Their use is mainly reserved for advanced disease stages or palliative care, when patients frequently report increased pain, often accompanied by sleep disturbances [94]. While opioids are effective in managing severe nociceptive pain, their prescription requires careful monitoring due to potential adverse effects, such as respiratory depression [108]. Their application is essential in selected cases but should always be carefully balanced against potential side effects.

(2) Neuropathic Pain

For neuropathic pain, medications including gabapentin, pregabalin, and tricyclic antidepressants are commonly prescribed [94,108]. Although evidence in ALS-specific neuropathic pain remains limited, these agents are applied according to established neuropathic pain management protocols [99].

(3) Spasticity

Effective management of spasticity and associated pain is a key component of symptomatic therapy in ALS. Antispastic agents such as baclofen and tizanidine may be used to reduce muscle stiffness and discomfort [16]. In cases of severe or refractory spasticity, intrathecal baclofen has shown greater efficacy than oral therapy, improving quality of life [1].

(4) Muscle Cramps

Muscle cramps are a common source of pain in ALS. They can be treated with quinine, benzodiazepines, magnesium, or carbamazepine [90]. Symptomatic treatment of muscle cramps also includes ensuring adequate hydration and treatment of electrolyte imbalances [16]. According to Pota et al. [94], quinine sulfate remains one of the most frequently used pharmacological treatments for muscle cramps in ALS. They report that 58% of surveyed ALS centers still prescribe quinine sulfate, despite ongoing concerns regarding its cardiotoxic and hematologic effects. Benzodiazepines, magnesium, and carbamazepine are prescribed in 40%, 25%, and 23% of centers, respectively. A relatively new therapeutic option for muscle cramps is mexiletine, a sodium channel blocker. In a multicenter, randomized, double-blind, placebo-controlled crossover trial, 20 patients received 150mg of mexiletine twice daily. Both the frequency and severity of cramps were significantly reduced compared with placebo. Eighteen out of twenty patients showed improvement, and no serious adverse event occurred [110].

(5) Cannabinoids

These agents are increasingly being studied for their analgesic and muscle-relaxant effects. Some studies’ reports are in line with our experience of positive effects on spasticity, pain, and mood. Clinical evidence remains limited at present [94].

2. Non-pharmacological treatment

Physical therapy is the mainstay of treatment of spasticity in ALS [1]. In contrast, non-pharmacological approaches to pain management represent an important but insufficiently studied aspect of multidisciplinary ALS care. The guidelines of the European Academy of Neurology (2024) recommend systematic pain assessment and supportive interventions such as physiotherapy, positioning, and splinting to address musculoskeletal complications, including frozen shoulder and joint pain. Despite the severe impact of pain on quality of life, evidence for the effectiveness of these interventions remains limited. A systematic review and meta-analysis by Papadopoulou et al. [111] found no statistically significant benefit of strength training, aerobic exercise, or manual therapy in reducing pain. Similarly, Rojas-López et al. [112] reported a lack of proven efficacy, emphasizing the ongoing shortage of clinical evidence for non-pharmacological pain management in ALS.

#### 2.2.6. Psychological Aspects in ALS

In the past, ALS care focused predominantly on physical impairments. More recent approaches emphasize an integrative view that includes the psychosocial and psychological well-being of both patients and caregivers [113,114]. Psychological, existential, and supportive factors are well recognized as essential for maintaining quality of life despite severe functional decline [115,116,117]. Studies have confirmed that satisfactory quality of life is possible even with significant physical impairments if psychosocial adaptation is supported [115,117,118,119,120].

##### Depression, Anxiety

Depression and anxiety are highly prevalent in ALS, negatively affecting quality of life [121,122]. In principle, these disorders are treatable with antidepressants, anxiolytics, and cognitive behavioral therapy. However, ALS-specific issues such as a high rate of suicidal thoughts, finding meaning in life despite severe physical limitations, and altered life perspectives require targeted psychotherapeutic approaches [123,124]. Although there is a high need, only a few clinical trials have evaluated psychotherapeutic interventions in ALS. Therefore, there is a lack of ALS-specific treatment programs dedicated to psychotherapeutic intervention programs dealing with the management of comorbid depression and anxiety in patients with ALS [121]. Clinical experience, however, indicates that psychotherapeutic support positively influences well-being and quality of life. Coping with the disease seems to play an important role in this context, which can be supported by psychotherapeutic interventions.

##### Coping Strategies

More recent research results indicate that well-being in ALS is not only determined by physical health but is strongly influenced by coping strategies. These strategies also affect the development of depression and anxiety [125,126,127]. ALS patients use different coping strategies, with problem-oriented coping being associated with better quality of life and emotion-oriented coping linked to lower levels of depression, as shown in the systematic review by Oh, An, and Park [128]. Other factors, such as sociodemographic and disease-related factors, also seem to have an influence on the coping strategies applied. Younger patients use different strategies compared with older patients [128,129,130]. Concerning gender specific differences, no consistent findings can be reported.

The current research provides inconsistent evidence on whether disease duration has an influence on coping behavior. While some authors report stable use of coping strategies throughout the course of the disease [126], others indicate changing coping behaviors depending on the disease duration [118,129,130]. The latter findings confirm our clinical experience. Coping with ALS should be understood as a process extending throughout the entire course of the disease for patients and caregivers. This opens opportunities for therapeutic interventions, as demonstrated in a recent study by Vandenbogaerde et al. [131].

Furthermore, studies highlight certain coping strategies as being particularly helpful. In ALS, spirituality and religiosity are described as providing psychological strength by preventing despair and finding meaning in the present situation [119,132].

##### Social Support

Social support plays a key role in coping with ALS. Patients actively seeking support has been identified as a leading coping strategy [133]. Experienced support significantly influences quality of life, with family, relatives, and caregivers serving as key providers [118,119,134]. Social support also seems to have an impact on depression severity [114,119] as well as on finding meaning in life [123], which is regarded as important for the use of adaptive coping strategies [134]. Clinical experience shows that maintaining autonomy throughout the disease course is an important need for ALS patients, closely linked to social support.

##### Caregiver Burden

ALS brings major challenges not only for patients but also for caregivers. Over the course of the disease, this often results in increasing psychological strain that can lead to depression and anxiety [135,136]. To help reduce this burden, the use and acceptance of technological devices such as powered wheelchairs or communication aids can support both caregivers and patients by enhancing autonomy and self-determination [120]. There is a broad consensus that the psychological well-being of caregivers must be addressed in ALS care to ensure adequate and psychotherapeutic support [136].

##### Cognitive and Behavioral Impairments

In about 10% of patients with ALS, cognitive, emotional, and behavioral changes indicative of frontotemporal dementia (FTD) can be found [137]. As recent studies have shown, such changes impair adaptive coping strategies, including humor comprehension [138]. They also significantly reduce quality of life, particularly in patients with behavioral or rapid cognitive decline. To address specific diagnostic complexities, specialized neuropsychological tools are being validated. So far, only a few standardized instruments like the Edinburgh Cognitive and Behavioral ALS Screen (ECAS) have been approved as recommended standard tools [139,140]. However, diagnostic limitations persist due to the scarcity of validated non-English or German-language instruments [141,142]. Since motor and speech impairments limit standard assessments, detailed observation and caregiver reports play a crucial role. Baumann et al. [143] describe this approach as an ongoing diagnostic process requiring careful differentiation between behavioral and anxiety-related symptoms [143]. In the early stages of ALS, neuropsychological assessments focus on detecting initial signs of cognitive impairment. Standard diagnostic tools include established tests such as the Test Battery for Attention Performance (TAP) [144], the Vienna Test System (WTS) [145], the Wechsler Memory Scale-Revised (WMS-R) [146], the Wechsler Adult Intelligence Scale–Fourth Edition (WAIS-IV) [147], and the Regensburg Verbal Fluency Test (RWT) [148]. When more pronounced deficits are suspected, or an initial screening is required, instruments such as the Clock-Drawing Test (CDT) [149], the Mini-Mental State Examination (MMSE) [150], the Montreal Cognitive Assessment (MoCA) [151], and the CERAD (Consortium to Establish a Registry for Alzheimer’s Disease) are commonly used.

Behavioral aspects and observations, including caregiver reports, are important criteria in the diagnosis of FTD. Diagnostic indicators include a decline in social behavior, affective flattening, and reduced disease awareness. Associated symptoms comprise behavioral disturbances, such as preservation, repetitive motor stereotypes, stimulus-bound behavior, speech and language disorders, primitive reflexes, incontinence, akinesia, rigidity–tremor, and hypotension [152].

Neuropsychological diagnostics related to FTD in the context of ALS are, therefore, less focused on aspects of psychomotor processing speed. Instead, greater emphasis is placed on other domains, such as verbal fluency [153]. Guidelines also emphasize the importance of early behavioral evaluation after confirmation of cognitive symptoms. Patients should be assessed early regarding verbal fluency, executive functions, and social behavior, while considering the limitations of psychomotor processing speed due to motor impairments [154].

The meaning of neuropsychological diagnostics becomes evident with respect to cognitive impacts on, for example, communication abilities, coping behavior, and quality of life in ALS. Although therapeutic options are limited due to the outlined limitations in ALS, the cognitive changes remain highly relevant in the process of applying interventions [115].

Active and early diagnostic assessment should be undertaken in specialized neuromuscular clinics by experienced neuropsychologists working within an interdisciplinary team, due to the complexity of differential diagnosis [113,115].

#### 2.2.7. Palliative Care

The management of ALS requires palliative care as a fundamental part, with a multidisciplinary approach, addressing the complex physical, psychological, and existential challenges of the disease. According to German Guidelines and the European Academy for Neurology (EAN), ALS care is by nature palliative, aiming to improve quality of life through the prevention and relief of suffering via early identification and treatment of physical, psychological, and spiritual problems [3,16]. Within this framework, “palliative” is not limited to end-of-life care but covers active and symptom-oriented management throughout all disease stages [155,156]. Palliative care should be introduced early in the disease course and continue throughout all stages, ensuring continuity of multidisciplinary support from diagnosis to end of life [8,16].

Palliative care in SLT focuses on maintaining communication and swallowing functions, counselling patients and caregivers regarding nutrition and ventilation decisions, and supporting autonomy and participation [3,10]. In respiratory care, early discussion of NIV and invasive ventilation options, including their implications for communication, autonomy, and quality of life, is essential [16,87]. The same applies to decisions about enteral feeding or “eating and drinking with acknowledged risks” [32,33].

Psychological and existential dimensions form another core element of palliative care in ALS. Studies emphasize the significance of coping strategies, social and spiritual support, and psychological interventions in maintaining quality of life and also contribute to reducing depression and anxiety [115,125,134].

In advanced stages, pharmacological symptom control, including opioid use for pain, becomes part of the palliative approach [94,108]. In addition to addressing patient needs, structured palliative interventions can also reduce caregiver burden and improve coordination, communication, and psychosocial support both in home and inpatient settings [157]. Overall, palliative care in ALS extends across all phases of the disease. This approach integrates symptom management, communication and decision support, psychosocial counselling, and advanced care planning within a patient-centered, multidisciplinary framework [3].

## 3. Conclusions

Rehabilitation in ALS is an essential and evidence-based part of multidisciplinary care. It focuses on preserving function, autonomy, and quality of life, since no curative treatment exists. The integration of physical, occupational, respiratory, speech and language, psychological, and palliative interventions allows for a comprehensive, patient-centered approach. This multidisciplinary framework addresses the complex and evolving needs of individuals with ALS. With the advancement of modern molecular and gene-based therapies, the role of rehabilitation in ALS remains important. As life expectancy is expected to improve, the focus on long-term motor function support, autonomy, and quality of life will be even more essential. In our opinion, rehabilitation remains underrated in daily clinical practice despite substantial evidence supporting its benefits. The existing literature summarized in this review strongly indicates that rehabilitative care deserves far greater attention, both in clinical implementation and scientific research. We strongly call for intensified neurorehabilitation research in ALS to establish stronger evidence for rehabilitative interventions and to advance quality of life as a central therapeutic outcome.

Current evidence supports moderate, individualized exercise programs, early respiratory therapy, and structured management of spasticity, pain, dysphagia, and communication disorders as key elements of symptomatic treatment. Psychological and social interventions, including caregiver support, play a pivotal role in maintaining emotional well-being and coping ability. These interventions also help reduce depression, anxiety, and caregiver burden. In conclusion, rehabilitation in ALS must be viewed as a dynamic and continuous process that integrates medical, functional, and existential dimensions. It thereby forms the cornerstone of patient-centered, multidisciplinary ALS care.

## Data Availability

No new data were created in this study.

## Abbreviations

The following abbreviations were used in this manuscript:

AAC	Augmentative and Alternative Communication
AI	Artificial Intelligence
ALS	Amyotrophic Lateral Sclerosis
ALSFRS-R	ALS Functional Rating Scale–Revised
BPI	Brief Pain Inventory
CDT	Clock-Drawing Test
CERAD	Consortium to Establish a Registry for Alzheimer’s Disease
EAN	European Academy of Neurology
ECAS	Edinburgh Cognitive and Behavioral ALS Screen
EDAR	Eating and Drinking with Acknowledged Risks
EAT-10	Eating Assessment Tool–10 Items
EMST	Expiratory Muscle Strength Training
FEES	Fiberoptic Endoscopic Evaluation of Swallowing
fALS1	Familial Amyotrophic Lateral Sclerosis Type 1
FDT	Frontotemporal Dementia
IDDSI	International Dysphagia Diet Standardization Initiative
I^2^	Statistical Heterogeneity Index in Meta-Analysis
MMSE	Mini-Mental State Examination
MoCA	Montreal Cognitive Assessment
NIV	Non-Invasive Ventilation
NMDA	N-Methyl-D-Aspartate Receptor
NSAIDs	Nonsteroidal Anti-Inflammatory Drugs
PCF	Peak Cough Flow
PEG	Percutaneous Endoscopic Gastrostomy
PES	Pharyngeal Electrical Stimulation
pCO_2_	Partial Pressure of Carbon Dioxide
RCSLT	Royal College of Speech and Language Therapists
RWT	Regensburg Verbal Fluency Test
SLT	Speech and Language Therapy
SNIF	Sniff Nasal Inspiratory Pressure
SOD1	Superoxide Dismutase 1
SWAL-QOL	Swallowing Quality of Life Questionnaire
TAP	Test Battery for Attention Performance
tcCO_2_	Transcutaneous Carbon Dioxide
VFSS	Videofluoroscopic Swallow Study
WAIS-IV	Wechsler Adult Intelligence Scale–Fourth Edition
WMS-R	Wechsler Memory Scale–Revised
WTS	Vienna Test System
